# Does poverty increase antibiotic prescribing rates through underlying health conditions? Ecological study using parallel mediation analysis

**DOI:** 10.1017/ash.2022.372

**Published:** 2023-01-30

**Authors:** Nato Tarkhashvili

**Affiliations:** Black Hills Special Services Cooperative, Pierre, South Dakota

## Abstract

**Objective:**

Effect of social determinants on antibiotic prescribing rates is poorly studied in modern literature. The objective of this study was to explore the effect of the prevalence of poverty (annual household income <$24,999) in each state on antibiotic prescribing rates in outpatient settings per 1,000 population through chronic health conditions (ie, prevalence of obesity, diabetes, and chronic obstructive pulmonary disease) while also adjusting for confounders (ie, prevalence of population aged ≥65 years and physician density in each state).

**Design:**

Ecological study.

**Participants:**

Entire US population.

**Methods:**

Prevalence estimates from all 51 states were used to calculate direct, indirect, and total effects of poverty on the rates of antibiotic prescribing through parallel mediation analysis using linear regression with chronic health conditions (obesity, diabetes, and chronic obstructive pulmonary disease) as mediators. I obtained these data from point-prevalence estimates of 2020 survey results from the Behavioral Risk Factor Surveillance System for rates on poverty, obesity, diabetes, chronic obstructive pulmonary disease, and population aged ≥ 65 years. I also used the Antibiotic Resistance & Patient Safety Portal for antibiotic prescribing rates per 1,000 population and the Association of American Medical Colleges database for the physician density per 100,000 population.

**Results:**

For every percentage increase in prevalence of poverty in each state, the antibiotic prescribing rate increased by 17.4 courses per 1,000 population (95% bootstrap confidence interval, 9.2–24.9) using indirect effects of poverty through mediators.

**Conclusions:**

Antibiotic stewardship programs should consider targeting social determinants of health along with underlying health conditions of patients being treated with antibiotics.

According to the Centers for Disease Control and Prevention (CDC) every year 2.8 million infections are caused by drug-resistant bacteria, and 35,000 people die from these infections.^1^ Treating infections becomes more and more challenging because antibiotic-resistant bacteria leave very few options to clinicians and are essentially untreatable.^1^ The problem is truly worrisome given the fact that new antibiotics are unlikely to emerge on the market, and old antibiotics are no longer effective against drug-resistant bacteria.^2^ The development of resistance to antibiotics is a natural process; however, using antibiotics in clinical practice accelerates the process and generates drug-resistant strains.^3,4^ Decades ago, antibiotic use was recognized as the strongest driving force for resistance development,^5,6^ and higher frequency of prescribing increases the chances of resistance development.^3,4^ Therefore, the modern discourse of antibiotic stewardship programs aims to reduce the amount of antibiotics prescribed.^7^ The goal of antibiotic stewardship has been to promote prescribing only when needed, at right time, at right doses, and at a right duration.^8^ In other words, it is intended to prevent inappropriate prescribing.

Inappropriate prescribing is a serious public health issue given that ∼30% of all antibiotics are prescribed inappropriately (eg, without indication).^9^ Inappropriate prescribing significantly increases the amount of antibiotics prescribed and therefore, increases the chances of resistance development.^9^ Antibiotic stewardship as a new initiative aiming to reduce antibiotic prescribing rates, has traditionally focused on physician behavior and organizational factors to reduce overall prescribing rates through preventing inappropriate prescribing.^10^

Although inappropriate prescribing may account for a significant chunk of antibiotics being prescribed, patient-specific factors may also contribute to high prescribing rates through appropriate prescribing mechanism, but this has not been studied recently. Scientific knowledge about patient-specific factors that may also increase or decrease antibiotic prescribing rates in clinical settings is limited. Situations may arise in which prescribers encounter patients with multiple underlying health conditions who may, in fact, benefit from antibiotics. For example, patients diagnosed with chronic obstructive pulmonary disease (COPD) may benefit from antibiotics during episodes of exacerbation.^11^ Alternatively, patients diagnosed with obesity or diabetes, who are predisposed to frequent bacterial infections, may also require antibiotic prescriptions at a higher rate compared to healthy patients without those conditions.^12,13^ Populations with higher prevalences of those conditions may also receive more antibiotics through appropriate prescribing mechanisms. In this context, the role of social determinants that “create” and propagate chronic health conditions also remains largely uncharacterized and underappreciated by public health community. This fact is reflected in the current discourse of antibiotic stewardship programs which focuses strictly on prescriber behavior without considering the broader problems.^9^ The goal of the current study was to characterize relationship between poverty and antibiotic prescribing rates through mediating effects of underlying health conditions (1) to evaluate the effects of underlying health conditions on prescribing rates and (2) to evaluate the effects of poverty on prescribing rate through mediators (ie, underlying health conditions).

## Methods

In the current study, I used ecological design to explore relationships between selected group of variables. The outcome variable for analysis was outpatient antibiotic prescriptions per 1,000 population for each state dispensed from community pharmacies in calendar year 2020. Because outpatient antibiotics dispensed reflects the number of antibiotic courses prescribed, I have used the term “antibiotic prescribing rate per 1,000 population.” Data on rates of outpatient antibiotics dispensed from community pharmacies per 1,000 population were obtained from the CDC website (IQVIA Xponent data).^14^

The independent variable “poverty” was defined as a prevalence of population with annual household income <$24,999 for each state per 100 inhabitants, and these data were obtained from Behavioral Risk Factor Surveillance System (BRFSS) survey results of 2020.^15^ This threshold for poverty was based on the 2020 Federal poverty level for a household with 3 members, which was $21,720; however, the average household size in 2020 was 2.53 according to the US Census.^16,17^ Thus, the poverty threshold for a household with 2.53 persons was calculated as $18,317, which was within the range of $0–$24,999 provided by the BRFSS. Mediating variable data, such as prevalence of COPD, diabetes, and obesity per 100 inhabitants for each state, were also obtained from BRFSS survey results of 2020.^15^

### Potential confounders

Confounders, such as physician density per 100,000 population and the prevalence of population aged ≥65 years per 100 inhabitants, have been included in the model. In previous studies, physician density has been shown to increase or decrease the services provided and improve access to care, although no study has specifically reported the impact of physician density on antibiotic prescribing rates.^18^

Prevalence of aging population was also considered a potential confounder based on previous research showing that elderly patients have higher rates of antibiotics prescribed.^19^ To account for those confounding effects, prevalence data for the elderly population (aged ≥65 years) for each state were obtained from BRFSS survey results for 2020, and the data on physician density were obtained from recent report published by the Association of American Medical Colleges (ie, number of practicing medical doctors per 100,000 population for each state in 2020).^15,20^ Although certain specialties have been shown to have higher prescribing rates than others, only 1 variable was included in the model to show all specialties combined per 100,000 population for each state.^21^

### Statistical analysis

**Fig. 1.**
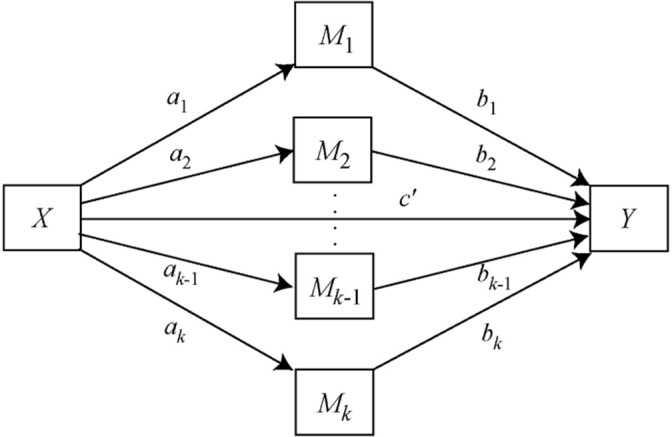
Parallel multiple mediator model.^31^

Mediation analysis was based on linear regression using the ordinary least squares method and was conducted using SPSS version 27 software (IBM, Armonk, NY) with the macro PROCESS (version 4.1). Parallel mediation analysis was conducted using model number 4, which allowed the selection of an independent variable (prevalence of poverty) along with dependent variable (antibiotic prescriptions dispensed per 1,000 population), multiple mediators (eg, prevalence of COPD, diabetes, obesity) linking independent and dependent variables, and several confounders (eg, prevalence of aging population and physician density per 100,000 population) (Fig. 1).^22^ The analysis was conducted using 5,000 bootstrap samples that no longer required normal distribution of scores nor the Sobel test.^22^ Because ecological design encompassed all 51 states including District of Columbia, no power analysis nor sample size calculations were made.

Simple mediation analysis usually involves analyzing data in 3 consecutive steps.^22^ First, relationships between independent and dependent variables are explored. Second, relationships between independent variable and each mediator are explored. Finally, relationships between mediators and dependent variables adjusted for independent variable are explored. In the current analysis, the first step was executed using multiple linear regression where independent variable (ie, prevalence of poverty) and was regressed against antibiotic prescribing rates while also adjusting for confounders (ie, prevalence of aging population and physician density per 100,000 population). In the second step, the relationships between independent variable (prevalence of poverty) and each mediator (ie, COPD, diabetes, and obesity) were explored while also adjusting for confounders (ie, prevalence of aging population and physician density per 100,000 population). In the third step, an independent variable (prevalence of poverty) was regressed against a dependent variable (antibiotic prescribing rates per 1,000) adjusted for all 3 mediators and confounders.

## Results

Descriptive statistics showed that average prescribing rate of antibiotics in outpatient setting was 619.86 per 1,000 population in 2020 with high variability among the states: West Virginia reported the highest rate of 974 courses per 1,000 population, and Alaska reported the lowest rate of 348 courses per 1,000 population (Table 1).

**Table 1.** Descriptive Statistics of Variables Used in the Study

Variable	Prescriptions per 1,000 Population	Prevalence of Poverty, %	Physician Density per 100,000	Prevalence of Population aged ≥65 y, %	Prevalence of COPD, %	Prevalence of Obesity, %	Prevalence of Diabetes, %
**US states**							
Valid	51	52	52	52	52	52	52
Missing	1	0	0	0	0	0	0
Mean	619.86	24.14	266.82	22.39	6.61	32.04	10.89
SE of mean	20.69	1.18	13.94	.31	.27	.56	.30
Median	618.00	22.15	248.20	22.45	6.20	31.70	10.70
SD	147.74	8.53	100.54	2.24	1.98	4.07	2.17
Minimum	348	14.7	163.6	15.9	3.7	24.2	7.5
Maximum	974	72.8	849.0	26.8	13.6	39.7	15.8

Note. COPD, chronic obstructive pulmonary disease; SE, standard error; SD, standard deviation.

The first step of mediation analysis indicated the existence of a strong linear relationship between the prevalence of poverty in each state and the antibiotic prescribing rate per 1,000 population (unstandardized β = 18.1; R^2^ = 0.39), indicating that for every percentage increase in prevalence of poverty in each state, the antibiotic prescribing rate increased by 18 courses per 1,000 population (*P* < .01) (Table 2). The second step in the analysis also indicated a statistically significant relationship between the prevalence of poverty and the prevalence of each mediator in the analysis. For every percentage increase in the prevalence of poverty adjusted for aging population and physician density, the prevalence of COPD increased by 0.2% (*P* < .01), the prevalence of diabetes increased by 0.29% (*P* < .01), and the prevalence of obesity increased by 0.34% (*P* < .01). All 3 models showed statistically significant relationships, with R^2^ values of 0.48, 0.64, and 0.45, respectively. The third step in mediation analysis also demonstrated strong relationships between all 3 mediators and the antibiotic prescribing rate, whereas the effect size of poverty decreased to a statistically nonsignificant level (unstandardized β = 0.64; *P* = .85).

**Table 2.** Elements of Parallel Mediation Analysis

Three Steps of Parallel Mediation Analysis	Parameter Estimate (unstandardized β)	95% CI	*P* Value	R^2^
**Step 1: Measuring relationship between independent variable (prevalence of poverty) and dependent variable (antibiotic prescribing rate per 1,000 population)**				0.39
Prevalence of poverty	18.1	11.0 to 25.1	<.01	
Prevalence of population aged ≥65 years	2.9	−12.9 to 18.7	.70	
Physician density	0.1	−0.3 to 0.4	.70	
**Step 2: Measuring relationship between independent variable (prevalence of poverty) and each mediator in the study (prevalence of poverty, obesity, COPD)**				
a) Outcome variable: prevalence of COPD				0.48
Prevalence of poverty	0.2	0.1 to 0.3	<.01	
Prevalence of population aged ≥65 y	0.3	0.1 to 0.5	.01	
Physician density	0.00	−0.0 to 0.0	.40	
b) Outcome variable: prevalence of obesity				0.45
Prevalence of poverty	0.3	0.2 to 0.5	<.01	
Prevalence of population aged ≥65 y	−0.1	−0.5 to 0.3	.60	
Physician density	−0.01	−0.03 to −0.01	<.01	
c) Outcome variable: prevalence of diabetes				0.64
Prevalence of poverty	0.3	0.2 to 0.4	<.01	
Prevalence of population aged ≥65 y	0.1	−0.1 to 0.3	.20	
Physician density	−0.0	−0.01 to 0.00	.20	
**Step 3: Measuring relationship between mediators and dependent variable (antibiotic prescribing rate per 1,000 population) adjusted for independent variable (prevalence of poverty) and confounders**				0.77
Prevalence of poverty	0.6	−6.3 to 7.6	.90	
Prevalence of COPD	25.5	6.7 to 44.2	<.01	
Prevalence of obesity	12.0	1.8 to 22.1	.02	
Prevalence of diabetes	28.0	5.1 to 50.9	.02	
Physician density	0.4	0.1 to 0.7	<.01	
Prevalence of population aged ≥65 years	−5.7	−17.4 to 6.0	.3	

Note. CI, confidence interval; COPD, chronic obstructive pulmonary disease.

### Direct, indirect, and total effects of poverty on antibiotic prescribing rate

In mediation analysis, total effects are the summary of direct and indirect effects of independent variable on dependent variable.^22^ In the current study, the data analysis showed that ∼96% of total effects were caused by indirect effects of poverty on antibiotic prescribing rates through mediators (ie, prevalence of COPD, diabetes, and obesity combined), with unstandardized β of total indirect effects of 17.4 of 18.1 (total effects) (Table 3). On the other hand, the direct effect of poverty on the antibiotic prescribing rate became negligible (unstandardized β = 0.64; *P* = .85). Although all 3 mediators exerted a statistically significant association on antibiotic prescribing rates in an indirect fashion, the effect of diabetes was the strongest (unstandardized β = 8.1; 95% bootstrap confidence interval, 1.5–16.4). Overall, this mediation analysis showed that the antibiotic prescribing rate increased by 17.4 courses per 1,000 population through mediators for every percentage increase in the prevalence of poverty in each state.

**Table 3.** Direct, Indirect, and Total Effects of Prevalence of Poverty on Antibiotic Prescribing Rates per 1,000 Population

Variable	Parameter Estimate (unstandardized β)	95% Bootstrap Confidence Interval	*P* Value
Total effects	18.1	11.0 to 25.1	<.01
Direct effects	0.6	−6.3 to 7.6	.90
**Indirect effects**			
Total	17.4	9.2 to 24.9	
COPD	5.2	0.6 to 9.6	
Obesity	4.1	0.2 to 8.9	
Diabetes	8.1	1.5 to 16.4	

## Discussion

Antibiotic prescribing is a complex process influenced by multiple factors such as physician’s behavior and experience, sex, patient expectations, organizational culture, etc.^23^ Although much has been written about those factors, there is a significant gap in the literature when it comes to documenting the impact of underlying health conditions on prescribing rates and the impact of social factors that inflate prescribing rates. In the current study, I considered antibiotic use from a mediation analysis perspective. There is a temporal relationship between the variables studied and antibiotic prescribing rates. This temporal relationship is a cornerstone of mediation analysis. Based on current knowledge, social determinants largely “create” chronic health conditions such as obesity, diabetes, and COPD.^24^ In other words, chronic health conditions cluster in the same areas due to the social and environmental conditions, a phenomenon called “syndemics.”^24^ For example, several researchers have documented that obesity is more prevalent among residents of poor communities.^25^ The same clustering has been observed for diabetes.^26^ Although COPD has been commonly associated with smoking, which is also more prevalent among underserved and poor populations, several researchers have documented a high prevalence of COPD among nonsmoking populations in rural and economically disadvantaged areas.^27^ Thus, the relationship between the poverty and mediators is well known. In fact, the chronic health conditions (eg, diabetes, obesity, COPD) examined in this study as mediators have been selected due to their high prevalence and syndemic association with the independent variable (poverty). The mediators in the current study have direct and linear relationship with outcome variables (eg, antibiotic prescribing rates) through direct or indirect mechanisms. For example, patients diagnosed with COPD may benefit from antibiotics and therefore, should receive antibiotics during episodes of exacerbation.^11^ On the other hand, patients diagnosed with diabetes and obesity may have a higher exposure rate to antibiotics because they are predisposed to bacterial infections, which may indirectly increase the need for antibiotic therapy.^12,13^ In current study, those relationships have been explored under steps 2 and 3 of mediation analysis. Each step demonstrated a strong, linear relationship with the outcome variable. In step 2, poverty was regressed against mediators, and in step 3 mediators were regressed against antibiotic prescribing rates. The relationship between prevalence of poverty and antibiotic prescribing rate was also statistically significant and showed a fully mediated process (96%), indicating that the entire effect of poverty on antibiotic prescribing was fully mediated by chronic health conditions (Table 3). This fact would allow the simplification of the conceptual model by omitting the effect of poverty on antibiotic prescribing rate because the direct effect of poverty prevalence on antibiotic prescribing rate was not significant (Table 3). The conceptual model can be presented as X (prevalence of poverty) → M (prevalence of COPD, obesity, and diabetes) → Y (antibiotic prescribing rates per 1,000 population).

The current study linear regression analysis for individual confounders (ie, physician density per 100,000 population and prevalence of aging population) did not show a statistically significant association with antibiotic prescribing rates, even though several studies have documented higher prescribing rates among elderly populations.^19^ One potential explanation is that the states with higher prescribing rates might have lower prevalence of elderly population due to lower life expectancy caused by poor socioeconomic status. No studies have explored the effect of physician density on antibiotic prescribing rates in communities. Therefore, it is hard to make valid conclusions based on the multiple regression analysis results presented in Table 2.

The current study had several strengths. These results clearly demonstrate strong, linear relationship between poverty and outpatient antibiotic prescribing rate with ∼39% of variability in prescribing explained by the prevalence of poverty in a state. I capitalized on the strengths of ecological studies and used community characteristics to explore relationships among the variables. Another strength of the study is that it has exposed the association of poverty with antibiotic prescribing through mediators. This association would have gone undetected if multiple regression analysis with prevalence of poverty had been used as one of the confounders because the effect of poverty becomes negligible due to the fully mediated process (Table 3). Thus, in the current study, I utilized the mediating effect of a factor, which would have been undetected in a regular regression analysis commonly used in public health research.

The scientific literature related to patient-specific factors leading to higher prescribing rates is limited. Only a handful of researchers have considered prescribing rates in relation to poverty, and all of them have noted that poor and underserved communities have higher rates of prescribing, although the causes underlying higher prescribing rates have not been fully investigated.^28^ Thus patient-specific factors, which are not currently on the agenda of antibiotic stewardship programs, must be investigated through the lenses of underlying health conditions and social determinants.

This study also had several limitations. Perhaps the most important limitation is the ecological design, which is subject to so-called ecological fallacy. Ecological fallacy prevents researchers from exploring individual-level data simply because, in ecological studies, the unit of analysis is a community (in this case a state).^29^ Thus, ecological studies are subject to numerous confounders that are not always taken into account.^29^ This study may also have been confounded by unknown confounders. Another limitation was the use of the prevalence of certain chronic medical conditions along with the prevalence of aging population and poverty using BRFSS data. The problem with BRFSS point-prevalence estimates is that quite often they overlap in 95% confidence intervals, which reduces the accuracy of prevalence estimates used in this analysis.^15^ Furthermore, I was unable to separate diabetes type 1 from diabetes type 2 in data analysis due to the way BRFSS questionnaires are structured; the BRFSS questionnaire asks a question about ever being told by healthcare provider that he/she has diabetes.^15^ Although variables in this study have been shown to have high validity and reliability, it is still possible that due to self-reported information collected by BRFSS, the rates used in this study under- or overestimated the true prevalence of medical conditions or household income.^15^

For the outcome variable in this study, the IQVIA Xponent data presented on the CDC website do not include outpatient antibiotics dispensed from the federal facilities nor inpatient medications dispensed. Thus, these data may underestimate the true amount of antibiotics dispensed from community pharmacies.^14^ Also, the rates presented in this study may have been influenced by the COVID-19 pandemic. And finally, IQVIA Xponent data used in the current study do not differentiate appropriately prescribed antibiotics from inappropriately prescribed ones.^14^

Current discourse regarding antibiotic stewardship does not consider patient-specific factors and may in fact penalize physicians or organizations with higher prescribing rates. For example, peer-to-peer comparison has been shown to reduce antibiotic prescribing rates among top prescribers.^30^ Although reductions in prescribing rates are desirable, the drawback of this approach in stewardship programs may be the fact that those comparisons do not consider underlying health conditions of patients being treated by individual physicians. Also, physicians with higher prescribing rates may have patients with multiple underlying health conditions that in fact, require antibiotic therapy. Assuming that top prescribers are the ones who prescribe inappropriately and asking them to reduce their prescribing may not be quite desirable approach without considering patient-specific factors. The process of standardization, which would consider patient-specific characteristics, has yet to occur in the National Healthcare Safety Network (NHSN).^7^ The standardized antibiotic administration ratio, usually used for facility comparisons proposed by the CDC, does not account for patient mix.^7^ On the other hand, looking at antibiotic prescribing process without considering a broader context of social determinants is also detrimental to stewardship programs because without improving social determinants of populations under care, prescribing rates are unlikely to be reduced.
